# Primary metastatic prostate cancer between prognosis or adequate/proper medical therapy

**DOI:** 10.1186/s12957-020-02111-3

**Published:** 2021-01-04

**Authors:** Mahmoud Mustafa, Honood Abu Rass, Mothafr Yahya, Khaleel Hamdan, Yazan Eiss

**Affiliations:** 1 Urology Department, Faculty of Medicine and Health Science, An-Najah National University, Nablus, West Bank Palestine; 2 Pathology Department, Faculty of Medicine and Health Science, An-Najah National University, Nablus, West Bank Palestine

**Keywords:** Prostate, Prostate cancer, Hormonal therapy

## Abstract

**Purpose:**

To define the efficacy of standard androgen deprivation therapy (ADT) in the treatment of metastatic prostate cancer (PCa).

**Materials and methods:**

Fifty patients with mean age of 70.48 ± 9.95 years old (range 52–87) who had metastatic PCa and received ADT between 2014 and 2019 were retrospectively evaluated. Median values of pre-therapeutic PSA and Gleason scores were 50 ng/ml (range 8–1201) and 8 (range 6–9), respectively. All patients received luteinizing hormone-releasing hormone (LHRH) analogue and anti-androgen. The patients were evaluated in terms of age, pre-therapeutic PSA serum levels, Gleason scores, presence of metastasis, number and percentage of cores involved, nadir PSA, time to nadir PSA, duration of ADT, and PSA at last follow-up. Multivariate analysis was used to define the factors which have impact on ADT response. The mean follow-up period was 13.87 ± 7.78 months, (range 2–32).

**Results:**

All patients showed reduction in serum PSA level after initiation of ADT, and the median value of nadir PSA was 1.12 ng/ml (range 0.02–50). The mean value of time to nadir PSA was 3.85 ± 1.57 months (range 2–7). The median value of PSA at last follow-up was 2 ng/ml (range 0.02–50.21). Multi-variant analysis showed that nadir PSA have a significant correlation with pre-therapeutic PSA, PSA at last follow-up, age, and Gleason scores (*p* < .05).

**Conclusion:**

Standard ADT is a feasible option in the treatment of metastatic PCa. Gleason scores, age, pre-therapeutic PSA, and PSA at last follow-up have significant impact on outcomes of ADT. Further studies of high number of patients with long-term follow-up including other chemo-hormonal therapy and androgen receptor blockers should be carried out to confirm and improve efficacy of ADT.

## Introduction

Prostate cancer (PCa) is one of the most common cancers in male. However, rates of detection of PCa vary widely across the world, with Europe and the USA detecting higher frequency than South and East Asia [1]. In China, the incidence rate is 1.6 cases per 100,000, while 119.9 cases per 100,000 in the USA [2]. In the USA alone, there will be an estimated 191,000 cases of PCa diagnosed in the year 2020 of which approximately 20% will be metastatic [3]. In the Middle Eastern population, there are variations in PCa incidence, progression, and prognostication compared to the Western population which may be due to significant difference in key molecular events [4]. African-American men have a higher risk of lethal PCa compared to European-American men [5]. Korean men differ from Western men in PCa aggressiveness with high grade and advanced stage in Korean men [6]. These national and geographic variations in PCa may mandate variation in therapeutic protocols of metastatic or confined PCa. The optimal management of PCa still remains controversial. However, hormonal therapy is the best choice for the treatment of patients with metastatic PCa. Hormonal therapy is also used as a neoadjuvant and/or adjuvant therapy in patients undergoing radical prostatectomy or radiotherapy. Furthermore, hormonal therapy is sometimes used as the primary treatment for localized PCa, especially in aged patients. Advances in upfront hormonal or chemo-hormonal therapy have driven a dramatic shift in treatment paradigms. At the same time screening practice, increased access to novel imaging modalities and globally aging population will drive increased rates of metastatic castration sensitive PCa. To conclude, ADT occupy an important place in the treatment of one of the most common cancers in male. There is a widespread agreement that ADT alone is no longer the standard treatment for patients with metastatic castration sensitive PCa [7, 8], but there is no consensus on the alternative protocols, may be due to a wide range of prognostic factors and variations in clinicopathological characteristics of PCa*.* Yamada et al. investigated the establishment of treatment strategy for metastatic PCa in patients with extremely high initial PSA level [9]. They found that serum PSA level at diagnosis is considered to be a useful prognostic factor for progression in localized PCa, but in metastatic cancer, it is not of such utility [9]. They concluded that high serum PSA level was associated with favorable response to vintage sequential ADT antiandrogen [9]. Herein, we investigated the efficacy of standard ADT in the treatment of primary advanced PCa in patients with high pre-therapeutic serum PSA level.

## Materials and methods

A total of 50 patients with mean of age 70.48 ± 9.95 years old (range 52–87) who were diagnosed to have primary metastatic PCa and received standard ADT between 2014 and 2019 were retrospectively evaluated. The median pre-therapeutic PSA serum level was 50 ng/ml (range 8–1201). The median value of Gleason scores was 8 (range 6–9). Forty-one (82%) patients had bone metastasis and 6 (12%) patients had lymph node metastasis and 3 (6%) patients with distant organ metastasis. All patients received ADT (LHRH analogous and anti-androgen) as a standard therapeutic protocol, and regular follow-up was applied. All patients were diagnosed by transrectal ultrasound-guided biopsy done before initiation of ADT due to high level of serum PSA and/or abnormal digital findings. The demographic characteristics of the study group in terms of age, smoking, and symptoms were illustrated in Table 1. At last follow-up, complete evaluation of the patients was done including PSA serum level, presence of metastasis, lower urinary tract symptoms, pain, and drugs used were summarized in Table 2. The oncologic characteristics of the patients including Gleason scores, number and percentage of cores involved, pre-therapeutic PSA value, nadir PSA, time to nadir PSA, and duration of HT.

**Table 1:** Demographic features of the patients

Variable	*N*, percentage (%)
Number of patients (*n*)	50
Age (years, %)	
50–59	8 (16%)
60–69	11 (22%)
70–79	20 (40%)
80–89	11 (22%)
Smoking history (*n*, %)	
Yes	31 (62%)
No	19 (38%)
Family history (*n*, %)	
Yes	3 (6%)
No	47(94%)
Intermittency (*n*, %)	47 (94%)
Nocturia (*n*, %)	37(74%)
Dysuria (*n*, %)	24 (48%)
Weak urine stream (*n*, %)	48 (96%)
Hematuria (*n*, %)	6 (12%)

*n* number of patients

**Table 2:** Oncologic features of the patients

Variable	Percentage (%)
Patient (*n*)	50
Gleason score (*n*, %)	
6	3 (6%)
7	15 (30%)
8	25 (50%)
9	7 (14%)
Site of metastasis (*n*, %)	
Bone	41 (82%)
Lymph node	6 (12%)
Organ	3 (6%)
Gleason scores (median, range)	8 (range 6–9)
Pre-therapeutic PSA (ng/ml) (median, range)	50 (8–1201)
Nadir PSA (ng/ml) (median)	1.1 (0.02–50)
Time to nadir PSA (month) (mean ± SD, range)	3.85 ± 1.6 (1–7)
Cores involved (mean ± SD, range)	5.39 ± 3.11 (1–11)
Percentage of cores involved (mean ± SD, range)	67.12 ± 27.14 (4–100)
PSA at last follow-up (ng/ml) (median, range)	1.95 (0.02–85)

*PSA* prostate-specific antigens, *n* number of patients, *SD* standard deviation

The patients were classified into two groups, and a comparison was carried out: group I was patients with nadir PSA serum level < 1 ng/ml; group II was patients with serum PSA level ≥ 1 ng/ml (Table 3). The patients who had increment in nadir PSA were also evaluated separately in terms of oncologic and demographic characteristics (Table 4). Multi-variant analysis and Pearson correlation tests were used to define the factors which may have impact on the response to ADT.

**Table 3:** Comparison between patients with PSA (< 1 ng/ml) and PSA (≥ 1 ng/ml)

Variable	Patient with PSA (< 1 ng/ml)	Patient with PSA (≥ 1 ng/ml)	*P*
Patients (*n*)	20	30	> 0.05
Age (year) (mean ± SD, range)	70.25 ± 8.17 (52–87)	70.06 ± 10.91 (52–89)	> 0.05
Pre-therapeutic PSA (ng/ml) (median, range)	26 (8–42)	82.50 (22–1201)	> 0.05
Gleason score (median, range)	7 (6–9)	8 (6–9)	> 0.05
Cores involved (mean ± SD, range)	5.19 ± 3.78 (1–12)	5.50 ± 2.75 (1–11)	> 0.05
Percentage of cores involved (mean ± SD, range)	56.88 ± 31.2 (4–100)	72.58 ± 23.4 (12.5–100)	> 0.05
PSA at last follow-up (ng/ml) (median, range)	0.12 (0.02–0.90)	4.64 (1.02–85)	> 0.05
Nadir PSA (ng/ml) (median, range)	0.08 (range 02–0.9)	2.75 (range 1.05–50)	> 0.05
Time to nadir PSA (months) (mean ± SD, range)	3.50 ± 1.42 (2–7)	4.03 ± 1.69 (1–7)	> 0.05
Bone metastasis (*n*, %)	17 (85%)	24 (80%)	> 0.05
Organ metastasis (*n*, %)	1 (0.05)	2 (6.66%)	0.04
Lymph node metastasis (*n*, %)	2 (10%)	4 (13.33%)	0.04
Follow-up (month) (mean ± SD, range)	13.81 + 9.07 (5–28)	13.17 + 6.57 (2–30)	> 0.05

*PSA* prostate specific antigen, *N* number of patients, *SD* standard deviation

**Table 4:** Oncologic and demographic characteristics of patients who showed increment in nadir PSA

Variable	*N*, percentage (%)
Patients (*n*, %)	15/50 (30%)
Age (years) (mean ± SD, range)	69.04 ± 10.42 (52–89)
Gleason score (median, range)	8 (6–9)
Metastasis (*n*, %)	
Bone	10 (66.67%)
Lymph node	4 (26.67%)
Lung	1 (6.66%)
Pre-therapeutic PSA (ng/ml) (median, range)	84.5 (20–1201)
Nadir PSA (ng/ml) (median, range)	2.5 (0.09–50)
Time to nadir PSA (month) (mean ± SD, range)	4.12 ± 1.75 (1–7)
Cores involved (*n*) (mean ± SD, range)	4.96 ± 2.27 (1–10)
Percentage of cores involved (mean ± SD, range)	70.87 ± 24.68 (12.5–100)
PSA at last follow-up (ng/ml) (median, range)	5.7 (1.1–85)
Follow-up (month) (mean ± SD, range)	13.69 + 6.26 (4–30)

*PSA* prostate specific antigen, *N* number of patients, *SD* standard deviation

### Statistical analysis

All data were presented as mean ± standard deviation (SD). IBM SPSS version20.0 software (SPSS Inc., Chicago, IL, USA) was used for the statistical analysis. A *p* value of > 0.05 was accepted as insignificant. Comparison between the parameter of subgroups was calculated by the use of *t* test and Wilcoxon signed-rank test. Pearson correlation and multi-variant analysis tests were used to measure the correlation of pairs of continuous variable.

## Results

All patients had significant reduction in the serum PSA level after initiation of ADT, and the median value of nadir PSA level was 1.2 ng/ml (range 0.02–50). Twenty patients (40%) achieved nadir PSA of < 1 ng/ml, and 30 patients had nadir PSA of ≥ 1 ng/ml. The mean value of time to nadir PSA was 3.85 ± 1.60 months (range 1–7): 14 patients (28%) achieved their nadir PSA after 3 months, 11 (22%) after 2 months, 9 (18%) after 4 months, 6 (12%) after 5 months, 6 (12%) after 6 months, 3 (6%) after 7 months, and 1 (2%) after 1 month. The median value of PSA at last follow-up was 1.95 ng/ml (range 0.02–85). The majority of the patients 31 (62%) aged above 70 years old and 8 patients (16%) were between 50 and 60 years old without any patient aged < 50 years old. Gleason scores were above 7 in 32 patients (64%), 15 patients (30%) have Gleason scores of 7, and only 3 patients (6%) have Gleason scores of 6. Comparison between patients with PSA less than or more than 1 ng/ml is shown in Table 3, and there were no significant differences between their variables. The mean ages of groups I and II were identical, i.e., 70.25 ± 8.17 and 70.06 ± 10.91, respectively (*p* > 0.05). The pre-therapeutic median of PSA values for both groups were 26 and 82 ng/ml, respectively, without statistically significant difference (*p* > 0.05). The median values of Gleason scores for group I and group II were 7 and 8, respectively (*p* > 0.05). The median values of PSA at last follow-up for group I and group II were 0.12 and 4.64 ng/ml, respectively (*p* > 0.05). The mean times to nadir PSA value in group I and group II were 3.5 ± 1.42 and 4.03 ± 1.69 months, respectively (*p* > 0.05).

Fifteen patients (30 %) who showed increment in nadir PSA level to > 1ng/ml had mean age of 69.04 ± 10.42 years old. Demographic and oncologic characteristics were illustrated in Table 4. The median values of pre-therapeutic PSA and Gleason scores were 84.5 ng/ml (range 20–1201) and 8 (range 6–9), respectively. The median nadir PSA value was 2.5 ng/ml (range 0.09–50). The mean time to nadir PSA was 4.12 ± 1.75 months, and the median value of PSA at last follow-up was 5.7 ng/ml (range 1.1–85). Ten patients (66.67%) had bone metastasis, and 4 (26.67%) had lymph node metastasis. Pearson correlation and multi-variant analysis showed that nadir PSA has significant correlation with pre-therapeutic PSA, PSA at last follow-up, age, and Gleason scores (*p* < .05).

## Discussion

Androgens are regarded as the fuel for hungry PCa [10]. Testosterone accounts for more than 90% of the systemic androgen function, and dihydrotestosterone (DHT) is its important variant [11]. The beneficial clinical effects of ADT in men with symptomatic metastatic PCa are rapid and dramatic [12]. Androgen deprivation therapy has been combined with number of upfront therapies of androgen receptor blockage including abiraterone acetate, enzalutamide, and apalutamide. There is paucity of direct comparator trial and there are no established predictive biomarkers to guide treatment selection, although tumor volume, oncologic features, metastasis to soft tissue, or organ and denovo/recurrent metastatic status are the key considerations for decision and regimes selection. For many patients who decline chemotherapy because of age, co-morbidities, or risk of toxicity of androgen receptors targeted agents, ADT is the preferred approach. Androgen receptor agents have general adverse events: abiraterone is given with prednisone to avoid toxicity related to mineralocorticoid activity. Enzalutamide and apalutamide are associated with an increased risk of falls and fracture [13, 14]. Apalutamide is associated with rash in 27% and hypothyroidism in 6.5%, and enzalutamide is associated with seizures 0.3% of patient [13, 14]. In our study group, the majority of the patients (60%) aged more than 70 years old, and many of them have co-morbid diseases, and because of side effects of chemotherapy and androgen blockage receptors, ADT was used as the standard protocol of therapy. Also, one of the distinguished features of our study is the high serum level of pre-therapeutic PSA (median 50 ng/ml) and all cases were primary cases; however, in literature, most of the primary diagnosed cases are either organ-confined or locally advanced cancer and those with high serum PSA level are mostly recurrent cancer after failure of the primary therapy. Up to knowledge, we could find only one study where the authors investigated the prognostic significance of extremely high initial PSA serum level in patients with primary metastatic PCa [9]. The range of PSA level is wider in patients with metastatic PCa than that in patients with localized PCa, and the clinicopathological characteristics in metastatic PCa are variable. The authors hypothesized that PCa with high serum PSA level may represent androgen receptor dependency and have high probability of mutation in androgen receptors [9].

We have some patients with pre-therapeutic PSA even > 1000 and > 500 ng/ml, as primary cases which is uncommon in the western countries. This study was a unique trial in detecting the effect of ADT in such primary patients with very high serum PSA level. We have found that all patients had significant reduction in PSA serum level after initiation of ADT within a short period. The mean value of nadir PSA for the study group was 1.2 ng/ml and the mean value of time to nadir PSA was 3.85 months, with 20 patients (40%) could achieve their nadir PSA < 1 ng/ml. Nadir PSA value is an important indicator of the success or good response of ADT in patients with metastatic PCa. As early as 1990, it was shown in small study that PSA decline after initiation of ADT predicted good survival in patients with metastatic cancer [15]. Patients who achieved reduction in PSA > 80% in the first month had longer progression-free survival compared to those who had reduction < 80% in PSA level [15]. In another study published by Fowler et al., PSA nadir < 1 ng/ml after initiation of ADT resulted in statistically significant longer time to biochemical recurrence [16]. They found that patients with nadir PSA < 1 ng/ml have significant difference and greater disease-free survival compared to patients who experienced a PSA nadir > 4 ng/ml [16].

The largest and the noteworthy study to look at PSA level after initiation of ADT for new diagnosed metastatic PCa was done by the Southwest Oncology Group [17]. This study showed that nadir PSA after 7 months of initiation of ADT is a strong predictor of median overall survival of about 75 months [17]. In contrast, patients with nadir PSA > 0.2 but < 0.4 ng/ml had median survival of 44 months [17]. Finally, patients who experienced PSA nadir at 7 months but > 4 ng/ml had median survival of only 13 months [17].. Serum testosterone level may be of value especially if measured at the time of nadir PSA after 7 months of imitation therapy to determine the strategies regarding the prognosis of the patient and the therapeutic agent which may be added [18]. In our study, we could measure the serum total testosterone level for 10 patients only; thus, it is difficult to make a definite comment regarding this issue. The average testosterone serum level was 0.03 ng/ml in the study group, i.e., below the castration level, even though the majority of these patients had poor oncologic features: Gleason scores were 8 for 6 patients, median pre-therapeutic PSA level was 46.7 ng/ml, 8 of these patients (80%) had bone metastasis, and the median value of nadir PSA was < 1 ng/ml. These findings show that ADT is an effective protocol for the treatment of metastatic PCa. We recommend the measurement of serum testosterone at nadir PSA to adjust the strategy protocol therapy. Most of our patients achieved good response of ADT, so changing the therapy protocol or adding a new agent were not necessary. These findings do emphasis on the variations in progression and prognostication of PCa; therefore, validation of western therapeutic protocols for the managements of PCa is recommend for other nations and societies [4].

We have divided the study group into two groups according to nadir PSA more or less than 1 ng/ml. We have found that those who achieved nadir PSA < 1 ng/ml showed no increment in their PSA above 1 ng/ml regardless to other oncologic features of the cancer like Gleason scores or pre-therapeutic PSA level, or presence of metastasis. We could not detect any significant difference between the two groups in their oncologic characteristic, although there was numerical difference between some variables: pre-therapeutic PSA serum levels were 26 and 82 ng/ml for group I and group II, respectively, without statistically significant difference and Gleason scores were 7 for group I and 8 for group II but no significant difference. Also, median values of PSA at last follow-up were 0.12 and 4.6 ng/ml, respectively, without significant difference between both groups. We expect this numerical difference may be statistically significant in a larger study group. On a multivariate analysis, we found that pre-therapeutic PSA, Gleason scores, and age had significant impact on nadir PSA level which is something expected. The majority of our patent (64%) had Gleason scores of ≥ 8. Only three patients had Gleeson scores of 6, but their average pre-therapeutic PSA was 41 ng/ml, two of them achieved nadir PSA of < 1 ng/ml, and one of them with pre-therapeutic PSA of 55 ng/ml had nadir PSA and PSA at last follow-up of 1.05 and 1.9 ng/ml, respectively. This confirms the optimistic outcomes of ADT in the treatment of metastatic PCa. Fifteen patients had Gleason scores of 7; three of them only had nadir PSA > 1 ng/ml. Seven patients with Gleason scores of 9: two of them could achieve nadir PSA < 1ng/ml, and one of them achieved PSA at last follow-up < 1 ng/ml. These findings support the efficacy of ADT even in patients with high-risk PCa.

In Table 4, we separately evaluated the patients (15/50, 30%) who showed increment in their nadir PSA, and we found that the median pre-therapeutic PSA was 84.5 ng/ml with the median Gleason scores of 8, the median nadir PSA was 2.5 ng/ml, and the median PSA at last follow-up was 5.7 ng/ml. This showed that ADT is also with good outcomes even in patients with poor oncologic features. This argument raises the question of the necessity for chemotherapy or androgen receptor blockers since the beginning. Thus, we may help the patients avoid the side effects and toxicity of such protocol especially those in advanced age. Does the geographic variation in the incidence of PCa mandate variation in response to strategies and protocol of therapy? Thus, proper or adequate therapy remains to be defined.

Smoking and PCa is another debatable issue with unclear relation and pathophysiology. In one of the largest systematic reviews regarding this issue, the authors concluded that smokers have a higher risk of PCa mortality and worse outcomes after treatment. Smoking cessation should be encouraged in men with or at risk of having PCa [19]. In our study, we have 31 smokers (62%) of the study group, and when we compared the smokers with nonsmokers we could not detect any significant difference between their oncologic features or outcomes. The median pre-therapeutic PSA for smokers and non-smokers were 43.5 and 56 ng/ml, respectively. The median value of nadir PSA for smokers and non-smokers were 0.95 and 1.35 ng/ml, respectively, and the mean time to nadir PSA for smokers and non-smokers were 4.07 and 3.5 months, respectively*.*

In literature, to maximize the benefit of hormonal therapy, a lot of debatable strategies especially for patients with biochemical recurrences including orchidectomy, LHRH agonist, combined hormonal therapy, LHRH antagonist, intermittent hormonal therapy, and peripheral androgen blockage are present [20–23]. Unfortunately, as the case of biochemical recurrence, our data remain limited on knowing and understanding the optimal treatment for these patients in this field.

## Conclusion

Standard androgen deprivation therapy is a feasible option in the treatment of metastatic PCa; however, the proper/adequate protocol of hormonal therapy remains to be defined taking into account the expected national or geographic variations in PCa. Gleason score, age, and pre-therapeutic PSA serum level have a significant impact on outcomes of ADT. Further studies with high number of patients and long-term follow-up should be carried out including other chemo-hormonal therapy agents and androgen receptor blockers to confirm our results and to improve the efficacy of ADT.

## Data Availability

Our data are available upon the request of the editorial office.

## Abbreviations

ADH: Androgen deprivation therapy
PSA: Prostate-specific antigens
LHRH: Luteinizing hormone-releasing hormone
PCa: Prostate cancer
SD: Standard deviation
*n*: Number
